# Development of a family functioning measure for Hispanic lesbian, gay, and bisexual adolescents

**DOI:** 10.3389/fpubh.2026.1970713

**Published:** 2026-10-07

**Authors:** Alyssa Lozano, Alexandra Torres Arsuaga, Frank R. Dillon, Richard C. Cervantes, Joseph Zolobczuk, Nikolas Montolio, Guillermo Prado

**Affiliations:** 1 School of Nursing and Health Studies, University of Miami, Coral Gables, FL, United States; 2 School of Counseling and Counseling Psychology, Arizona State University, Tempe, AZ, United States; 3 Behavioral Assessment Inc., Beverly Hills, CA, United States; 4 YES Institute, Miami, FL, United States

**Keywords:** adolescents, family functioning, gay, bisexual, Hispanic, lesbian, measure development

## Abstract

**Introduction:**

Hispanic lesbian, gay, and bisexual (LGB) adolescents experience elevated risks for HIV-related risk behaviors, drug use, and mental-health problems. These problems may be impacted by various family functioning behaviors (e.g., communication, parental involvement). However, existing family functioning measures may not adequately capture the culturally shaped, identity-specific family processes relevant to this population. Thus, we iteratively developed a user-informed measure of family functioning for Hispanic LGB adolescents.

**Methods:**

The study was conducted across phases from February to December 2025: (1) focus groups with Hispanic LGB adolescents to identify domains of family functioning and inform an initial item pool, (2) a modified Delphi panel of experts and community partners to evaluate feasibility and validity, and (3) cognitive interviews with Hispanic LGB adolescents to assess face validity.

**Results:**

Focus groups identified five domains of family functioning: (1) communication about sexual orientation and identity, (2) emotional safety and selective disclosure in the family, (3) caregiver misattunement and invalidating communication, (4) cultural expectations and identity expression in the family, and (5) parental involvement, monitoring, and shared family life. The Delphi process refined an initial 32-item pool to 22 items, improving clarity, specificity, and cultural congruence. Cognitive interviews revealed that the developed items provided strong evidence of face validity and relevance; adolescents also provided suggestions for refinements that focused on identity expression and conditional acceptance. The final measure includes 23 items.

**Conclusions:**

This study produced a culturally responsive family functioning measure grounded in Hispanic LGB adolescents' lived experiences.

## Introduction

Lesbian, gay, and bisexual (LGB) adolescents experience elevated risks for human immunodeficiency virus (HIV)-related outcomes, drug use, and mental health problems, such as depression and suicidality, compared to their heterosexual peers (1). For example, LGB adolescents were more than four times as likely to attempt suicide and twice as likely to use drugs as their heterosexual peers (1). LGB adolescents who are sexually active are also less likely to use a condom during sex than their heterosexual peers (39.9% vs. 56.1%) (1). These disparities are especially pronounced for adolescents who are also members of racial and ethnic minority groups (2, 3). Hispanic LGB adolescents are of particular concern because they face the combined effects of sexual minority stress and the contextual pressures associated with growing up in Hispanic families and communities, including cultural expectations about family loyalty, traditional gender roles, and disclosure of stigmatized identities (4, 5). Consequently, Hispanic LGB adolescents report disproportionately higher rates of adverse health outcomes such as suicidality and illicit drug use relative to their LGB peers that do not identify as Hispanic (6). This pattern may reflect, in part, contextual pressures within Hispanic families, including unaffirming parental attitudes and family rejection that have been linked to poorer mental health, drug use, and sexual risk outcomes (4, 7, 8). For example, nearly twice as many Hispanic LGB adolescents (26.8%) report lifetime drug use compared to LGB adolescents that do not identify as Hispanic (1). Moreover, Hispanic LGB adolescents face higher rates of HIV, lower rates of HIV testing, and higher rates of adverse mental health outcomes such as depression, compared to LGB adolescents that do not identify as Hispanic (9–11). These outcomes do not occur in isolation: adolescents who use drugs and have mental health problems are more vulnerable to HIV acquisition and vice versa (12–14).

Family functioning, broadly defined as the functioning of an adolescent's family system has long been recognized as a key predictor of adolescent health (15, 16). More specifically, positive family functioning components such as family effective communication, parental involvement, and proper monitoring of peers are associated with lower levels of sexual risk behavior, internalizing symptoms, and drug use among adolescents (16–19). Conversely, family conflict is linked to greater internalizing symptoms, suicidality, and drug use (19). Family functioning may be especially consequential for Hispanic LGB adolescents because family processes do not unfold in a cultural vacuum. Hispanic cultural values such as *familismo*, familial obligation, and respect for family harmony may shape the meaning of parental responses to a young person's sexual orientation, and in turn impact family functioning as a whole (5, 8, 20). Furthermore, disclosure of a sexual minority identity can create a period of adjustment for families in which parents and adolescents negotiate new expectations about communication, privacy, boundaries, and support (21, 22). These processes may influence health directly and may also shape broader protective resources, including emotional support and stable caregiving.

Although family functioning has been widely studied in Hispanic adolescent health research more broadly, available measures were not developed specifically for Hispanic LGB adolescents and therefore do not capture the full range of culturally and developmentally relevant experiences that matter for this population (23). Existing family functioning measures primarily assess general parenting or household processes such as communication, involvement, and monitoring, which, although important, may not capture identity-specific dynamics relevant to Hispanic LGB adolescents (19, 24). For instance, these measures often overlook disclosure, acceptance, and culturally shaped family responses. Emerging evidence suggests that these limitations extend beyond conceptual concerns to include psychometric limitations. Lozano et al. (25) found that commonly used family functioning measures demonstrated measurement noninvariance across Hispanic LGB and heterosexual adolescents, indicating that scores may not reflect equivalent constructs across groups and potentially limiting construct validity. For example, a Hispanic family may appear supportive in broad parenting terms while still struggling with communication about sexual orientation, monitoring of the adolescent's activities and peer relationships, or parental involvement following disclosure. Complementing these findings, Lozano et al. (26) found that Hispanic LGB adolescents questioned the face validity of existing measures, noting that items did not consistently reflect their lived experiences. In Hispanic families, these dynamics may also reflect active negotiation around cultural values, religious beliefs, and social expectations about sexual orientation (22). A measure intended to support psychological research and practice with Hispanic LGB adolescents therefore needs to account for both general family functioning and the culturally specific context in which that functioning occurs for Hispanic LGB adolescents.

Family and adolescent research increasingly recognizes that face validity depends on whether a measure accurately reflects the experience of the population being studied, rather than simply applying a general measure to a new group without adaptation (27–29). In the case of Hispanic LGB adolescents, this means that a valid measure should be grounded in the words, meanings, and priorities of adolescents themselves, and should reflect the family and cultural processes that are most salient to their lived experiences. User-centered and qualitative approaches are especially well-suited to this task because they can identify domains that may be underrepresented in existing scales and can support the development of items that are understandable, relevant, and acceptable to youth (30–33). These approaches are also consistent with best practices in measure development, which emphasize item generation from lived experience, expert review, cognitive testing, and iterative refinement (27, 31, 34).

Prior measurement development research in LGB populations demonstrates the value of these approaches (35–37). For example, the development of the Sexual Minority Stress Inventory showed that a rigorous, multi-phase process combining qualitative inquiry, expert review, and psychometric testing can produce a stress minority measure that is both developmentally appropriate and psychometrically sound (36). Similarly, measure development work in transgender and gender-diverse youth has highlighted the importance of clearly defining constructs, distinguishing them from related but nonidentical phenomena, and ensuring that items are inclusive of identities that may otherwise be excluded from conventional measures (37). These studies demonstrate that when a construct is theoretically important but not yet well-measured, a careful development process can yield a tool that advances both psychological research and intervention science. This study was designed in this tradition.

The present study describes the user-centered and Delphi method-driven, iterative development of a measure of family functioning for Hispanic LGB adolescents that is culturally congruent, developmentally appropriate, and responsive to the experiences of Hispanic LGB adolescents themselves. This design follows best practices in scale development by ensuring that item content is grounded in participant experience while also being refined through expert input and direct feedback from the target population (31, 34, 36). The project is grounded in the view that family functioning for Hispanic LGB adolescents is not fully captured by existing generic family measures because the meaning of family communication, involvement, and monitoring may differ when viewed through the intersecting lenses of sexual orientation and Hispanic cultural context. This measure is intended to capture the family-level processes that may shape health disparities and resilience among Hispanic LGB adolescents. This includes how families communicate about sexual orientation, respond to disclosure, express support or rejection, and navigate culturally specific expectations around loyalty, respect, and belonging. In doing so, this measure aims to provide a stronger tool for examining how family dynamics correspond to HIV-related outcomes, drug use, and mental-health problems in this population.

## Materials and methods

### Study design and overview

We developed a measure of family functioning for Hispanic LGB adolescents by conducting a psychometric study using a user-centered design and modified Delphi process (38). The study was conducted in three phases from February to December 2025 and an overview is provided in Table 1. In Phase 1, we conducted focus groups with Hispanic LGB adolescents to generate preliminary content domains and an initial item pool grounded in their lived experiences. In Phase 2, we convened a modified Delphi panel of community partners and experts in LGB health and culturally congruent measurement to rate item validity and feasibility, refine item wording, and establish consensus on a candidate item set. In Phase 3, we conducted cognitive interviews with Hispanic LGB adolescents to assess evidence of face validity, comprehensibility, and cultural relevance making final refinements to produce the measure in both English and Spanish versions. This design leveraged information that Hispanic LGB adolescents with lived experiences provided directly, along with input from community partners and experts in culturally congruent measure development for LGB and Hispanic populations. All study procedures were approved by the University of Miami Institutional Review Board.

**Table 1:** Overview of study design.

Phase	Purpose	Participants	Procedures	Data analysis
Phase 1: focus groups	Identify salient domains of family functioning and generate an initial item pool.	14 Hispanic LGB adolescents across three focus groups.	Focus groups explored family functioning experiences and reactions to existing family functioning measures.	A general inductive approach was used to identify themes. Multiple analysts coded transcripts, developed a codebook, and reached consensus through discussion.
Phase 2: modified Delphi panel	Refine candidate items and establish expert consensus regarding item validity and feasibility.	Six panelists (three academic researchers and three community members from LGBTQ-serving organizations).	Panelists completed two rounds of REDCap ratings and participated in two Zoom meetings to discuss item content, clarity, and cultural relevance.	Median validity and feasibility ratings, mean absolute deviation, and the A6R consensus criterion were used to guide item retention, revision, or exclusion.
Phase 3: cognitive interviews	Assess face validity, comprehensibility, and cultural relevance of the refined items.	13 Hispanic LGB adolescents.	Individual cognitive semi-structured interviews used think-aloud and probing techniques to evaluate item understanding and relevance.	A general inductive approach was used to analyze feedback on item clarity, relevance, and cultural appropriateness. Two analysts coded transcripts and reached consensus.

### Participants and recruitment

#### Phase 1: Focus group participants

In Phase 1, we recruited 14 Hispanic LGB adolescents to participate in focus groups (Focus Group 1, *n* = 5; Focus Group 2, *n* = 5; Focus Group 3, *n* = 4). Participants were recruited from organizations that serve adolescents who identify with a minoritized sexual orientation or identity, social events attended by such adolescents, social media, posting electronic flyers on community partner's websites, and through word of mouth (i.e., snowball sampling).

Adolescents were eligible to participate if: (1) they were between the ages of 13–19; (2) they reported a non-heterosexual identity, same-sex or both-sex attraction, and/or same-sex or both-sex behavior; (3) they were of Hispanic origin as defined by having one parent born in a country within Latin America; and (4) minor adolescents (ages 13–19) had disclosed a non-heterosexual sexual orientation or identity to at least one parent. Adolescents were excluded if the parent and/or adolescent was unwilling or unable to provide informed consent and/or assent.

#### Phase 2: Delphi panel members

The Delphi panel was convened through the principal investigator's professional and community networks and comprised individuals with expertise relevant to the development of culturally congruent measures for Hispanic LGB adolescents. The panel included six members: three academic researchers with expertise spanning Hispanic populations, LGB populations, and culturally congruent measure development and psychometrics, and three community members from LGB-serving organizations with direct experience working with Hispanic LGB adolescents. Panelists were purposively selected to ensure representation of both community-based perspectives and methodological and substantive expertise in sexual orientation, Hispanic populations, and measurement development.

#### Phase 3: Cognitive interview participants

Phase 3 used the same inclusion criteria and recruitment procedures as Phase 1. We recruited 13 Hispanic LGB adolescents through LGBTQ-serving community organizations, social media platforms, and referrals from Phase 1 participants. Of the participants enrolled in Phase 3, nine had previously participated in Phase 1 focus groups, providing continuity and allowing for member checking of the measure's development across phases.

### Phase 1: Focus groups

#### Procedures

Adolescents first completed an eligibility screener to verify the inclusion criteria. Eligible adolescents were informed about the study and their participation in a focus group discussion.

For adolescents under 18 who expressed interest, parents were contacted to review the study's objectives and provide informed consent; adolescent assent was obtained. For adolescents aged 18 or 19, consent was obtained. Focus groups explored salient dimensions of family functioning for Hispanic LGB adolescents, guided by questions examining family roles within Hispanic culture and the nature of parent–adolescent relationships (see Supplemental Table 1 for sample questions). To identify gaps in existing measurement approaches, participants reviewed items from established family functioning scales assessing constructs such as parent–adolescent communication, parental monitoring, parental involvement, and positive parenting, with each construct presented separately to ensure clarity. Participants were compensated $40 for their time in completing the focus group. Focus groups were conducted online via Zoom (39), lasted approximately 1 h, and were audio recorded. Recordings were de-identified, transcribed verbatim, reviewed for accuracy, and securely stored on University of Miami servers. Transcripts were uploaded to NVivo (40) for qualitative analysis.

#### Data analysis

Transcripts were analyzed using a general inductive approach (41) to identify salient components of family functioning among Hispanic LGB adolescents. The analysts (the first author and research assistants) independently coded an initial subset of transcripts to develop an emergent, data-driven codebook (42), which was then applied systematically across all transcripts. Participant responses were coded, with regular meetings held to discuss discrepancies, refine code definitions, and reach consensus. NVivo software was used to manage transcripts, organize codes, and link coded segments to broader thematic categories. The resulting themes directly informed the development of the initial pool of candidate items for the family functioning measure.

### Phase 2: Modified Delphi panel

#### Procedures

Following completion of Phase 1 analyses and the generation of an initial pool of candidate items, Phase 2 employed a modified Delphi procedure (34) to refine item content and establish expert consensus. Once informed consent was obtained, panelists completed two rounds of independent ratings of item validity and feasibility using structured surveys administered via REDCap (43), hosted on secure University of Miami servers. Validity ratings reflected the extent to which items were supported by existing evidence or professional consensus, whereas feasibility ratings reflected anticipated clarity, comprehensibility, and appropriateness for adolescent self-report. Validity and feasibility were rated on a nine-point scale (1 = *low*, 9 = *high*). After each round of ratings, summary statistics were reviewed to identify areas of agreement and divergence. Two videoconference meetings were then held via Zoom (39), each lasting approximately 90–120 min, to discuss items with discrepant ratings, address wording and content concerns, and identify potential gaps. Panelists were compensated $280 for participation in each meeting. Guided discussion prompts focused on the cultural relevance of item language, the extent to which items captured family dynamics and disclosure-related processes for Hispanic LGB adolescents, clarity and inclusivity of wording, integration of cultural context and family history, and the utility of items for identifying areas in need of intervention or support (see Supplemental Table 2). Meetings were audio recorded, and detailed notes were taken to document feedback. Based on summary statistics and panel feedback from each meeting, items were revised and redistributed for subsequent ratings, resulting in a refined set of items advanced to Phase 3.

#### Data analysis

Panelists' validity and feasibility ratings were summarized using the median as the primary indicator of central tendency, with the mean absolute deviation from the median used to characterize dispersion across ratings. Decisions regarding item retention were guided by these summary statistics. Items were advanced if they demonstrated strong content support, defined as median validity ratings between 7 and 9 and median feasibility ratings between 4 and 9, allowing for the inclusion of items that were conceptually sound but potentially amenable to further refinement. Agreement among panelists was evaluated using the A6R criterion, whereby consensus was defined as all six ratings falling within a three-point range of the median (e.g., 7–9 or 6–8). Items with median validity ratings below 7, median feasibility ratings below 4, or insufficient agreement were excluded from further consideration.

### Phase 3: Cognitive interviews

#### Procedures

Phase 3 employed one-on-one semi-structured cognitive interviews with Hispanic LGB adolescents to refine the developed measure and assess evidence of its face validity. Recruitment, screening, and consent/assent procedures mirrored those used in Phase 1.

Interviews were conducted online via Zoom, audio recorded, and transcribed verbatim. Cognitive interviewing techniques (31, 32) were used to examine how participants understood, mentally processed, and responded to the refined items. Participants were instructed to “think aloud” as they reviewed each item, describing its relevance to their own family experiences and commenting on clarity, appropriateness, and cultural fit. The interviewer used probing questions to elicit detailed feedback on item comprehension and meaning (see Supplemental Table 1).

Participants were also asked to identify any aspects of family functioning that they felt were important but not adequately captured by the existing items (see Supplemental Table 3).

Interviews lasted approximately 30 min. Participants were compensated $40 for their time in completing the interview. Recordings were transcribed verbatim, de-identified, reviewed for accuracy, and securely stored on University of Miami servers. Transcripts were uploaded to NVivo (40) for qualitative analysis.

#### Data analysis

Data from Phase 3 were analyzed using a general inductive approach consistent with the analytic strategy applied in Phase 1 (41), with analytic attention focused specifically on participants' feedback regarding the developed family functioning items. Transcripts were reviewed iteratively to identify patterns related to item clarity, relevance, and cultural appropriateness. Two analysts (the first and second author) independently coded transcripts using a data-driven codebook, applied codes across all interviews, and met regularly to discuss discrepancies and achieve consensus. NVivo software (41) was used to manage and organize the data and to support the refinement of thematic categories. Findings from this phase were used to inform additional revisions to the measure prior to finalization.

### Rigor and trustworthiness

Several strategies were used to enhance rigor and trustworthiness across study phases.

Prior to coding, the research team engaged in reflexivity to identify positionalities, assumptions, and potential sources of bias (27). Credibility was supported through member checking, whereby preliminary themes were shared with participants for feedback (44, 45), and through peer debriefing with the Delphi panel. Investigator triangulation was achieved through the involvement of multiple trained researchers in data analysis, and detailed documentation of analytic decisions supported thick description. The principal investigator, who conducted all focus groups and interviews, identifies as a Hispanic heterosexual cisgender woman with extensive experience working with Hispanic LGB adolescents; additional analytic and advisory perspectives were provided by coders and panelists who identified as LGB. Prior to data collection and analysis, team members discussed their positionalities and worked to bracket preconceptions to remain grounded in participants' perspectives (46).

## Results

### Phase 1 focus groups

The focus group participants were, on average, 16.7 years old (*SD* = *1.8*). Most participants were assigned female at birth (71.4%). In terms of gender identity, seven participants identified as female (50.0%), four as male (28.6%), one as transgender (7.1%), one as gender-fluid (7.1%), and one reported they did not know (7.1%). Half of the focus group participants were born in the United States (50.0%) while the other half were born in Latin American countries (e.g., Nicaragua and the Dominican Republic). Reported sexual orientation was diverse: one participant described themselves as gay (7.1%), four as lesbian (28.6%), four as bisexual (28.6%), and one as polysexual/omnisexual/sapiosexual/pansexual (7.1%). One participant reported they had not figured out or were in the process of figuring out their sexuality (7.1%), one said they do not think of themselves as having sexuality (7.1%), and two participants responded “don't know” (14.3%). Full participant demographics can be found in Table 2.

**Table 2:** Adolescent demographics.

Variable	Aim 1	Aim 2
	Focus groups	Interviews
	*n* = 14	*n* = 13
Mean (SD) or *N* (%)		
Age	16.7 (1.8)	16.7 (1.5)
Sex assigned at birth		
Male	4 (28.6)	3 (23.1)
Female	10 (71.4)	10 (76.9)
Gender		
Male	4 (28.6)	2 (15.4)
Female	7 (50.0)	9 (69.2)
Transgender	1 (7.1)	2 (15.4)
Gender-fluid	1 (7.1)	0
Don't know	1 (7.1)	0
Country of birth		
US	7 (50.0)	10 (76.9)
Outside of the US	7 (50.0)	3 (23.1)
Sexual orientation		
Gay	1 (7.1)	2 (15.4)
Lesbian	4 (28.6)	6 (46.2)
Bisexual	4 (28.6)	4 (30.8)
Polysexual, omnisexual, sapiosexual, or pansexual	1 (7.1)	0
Have not figured out or are in the process of figuring out sexuality	1 (7.1)	1 (7.7)
Do not think of self as having sexuality	1 (7.1)	0
Don't know the answer	2 (14.3)	0

Five interrelated themes characterized family functioning among Hispanic LGB adolescents. The themes reflect how communication about sexual orientation, emotional safety in the family, caregiver attunement, cultural expectations, and everyday family involvement intersect to shape Hispanic LGB adolescents' experiences. Importantly, the five themes identified from the focus groups were used to organize participants' perspectives on salient aspects of family functioning and to guide subsequent item development; they were not prespecified as subscales of the resulting measure. Illustrative quotes and corresponding developed items can be found in Table 3.

**Table 3:** Focus group themes, illustrative quotes, corresponding items, and Delphi panel results.

Focus group findings			Delphi panel results	
Theme	Illustrative quotes	Corresponding items	Round 1	Round 2
1. Communication about sexual orientation and identity	“*I think it does vary depending on the topic because at least with my primary caregiver, certain things are taboo, you don't really talk about it, usually regarding sex or sexual orientation or even gender, we don't talk about it, or even relationships regardless if it's with a boy or if it's with the same sex.” * *“It's the fear of being judged or saying the wrong thing and getting–not the hand or recommended, just saying something and then they don't agree and then it's just weird or awkward or you just don't feel comfortable.” * *“I think that depends a lot on the family because I know parents that are very–they take the topic very taboo. Then there are others, like mine, that they‘re very, I don't want to say liberal, but very open. They're okay. And they joke around with it, with the topic… So I think it depends a lot on the family. There are some that you can talk better about it.” * *“Yeah. It definitely was. I was embarrassed to tell her about it because of certain reasons. Like you guys say, Latin parents and stuff, and you just don't know how they'll react. But I eventually did it and stuff. It wasn't too bad.”*	I feel comfortable talking to my primary caregiver about my sexual orientation without feeling judged or self-conscious.	O	✓
		I feel comfortable discussing my beliefs about LGBTQIA+ culture with my primary caregiver without feeling judged or self-conscious.	O	X
		I feel hesitant to ask my primary caregiver for what I want or need when it relates to my sexual orientation.	O	✓
		My primary caregiver doesn't believe what I am saying regarding my sexual orientation.	O	X
		I avoid discussing certain topics, such as my sexual orientation, with my primary caregiver.	✓	✓
		My primary caregiver sometimes makes comments about LGBTQ+ people that they may not intend to be hurtful, but that still upset me.	O	O
2. Emotional safety and selective disclosure in the family	“*I feel like with your friends, actually, you talk to your friends and stuff, and they would give you better advice because they wouldn't treat you like a parent. They would be more chill about it and give you more advice because for a parent, they'd probably freak out and scream, freak out, get you in trouble, and your friends just don't really do that. They handle it in different ways than your parents.” * *“…there are certain topics I can go to my primary caregiver for, but there are other topics that I can't.” * *“…Because you could bring the topic but then it's different thing saying certain words or certain phrases. Maybe you could bring the topic about painting your nails…but then another thing would be saying specifically that you want to paint your nails. So two different things, there's the topic and then there's what you say and the bubble of the topics.” * *“I feel like it's just you have to be careful in general of what you say and what you don't say. You have to be careful with your words or what you let out. Something that you don't want them to find out, you have to be careful about that.”*	I sometimes feel afraid to ask my primary caregiver for what I need.	O	✓
		I talk to my friends about my problems before going to my primary caregiver because I worry they might react negatively.	X	—
		I sometimes say things to my primary caregiver that I later regret.	X	—
		I'm careful about what I say to my primary caregiver so I don't disrespect them.	O	✓
		There are certain things I don't tell my primary caregiver because I don't want them to find out.	✓	✓
		I feel more comfortable talking to one of my parents than the other because I'm concerned about how each might react differently.	X	—
3. Caregiver misattunement and invalidating communication	“*Or sometimes they're just neglectful. So, you find it hard to discuss problems with them because they don't really pay attention or they don't care.” * *“Hispanic parents are good listeners, but sometimes they don't really understand. So I think for that question, it could be that my primary caregiver is always a good listener but may not understand. Because they do listen, but they have a hard time understanding certain things.”*	My primary caregiver listens attentively and makes an effort to understand my perspective.	X	—
		My primary caregiver tends to focus more on their own priorities (e.g., grades in school) than on what matters to me.	✓	O
		Whenever I express my feelings, my primary caregiver makes me feel uncomfortable or bothered.	O	✓
	“*…When I'm feeling frustrated or I'm angry, I cry to get my emotions out of the way. And they'll be like, ‘Why are you crying?' or, ‘Nobody's hit you. Why are you so upset?' or, ‘Oh, crying isn't going to solve your issues.”' * *“…Sometimes family members compare you to other family members. So they'll be like, ‘why are you not good like your cousin or, like, your brother or your sister?”'*	My primary caregiver makes comments that they may not intend to be hurtful, but that still make me feel misunderstood or dismissed.	O	✓
		My primary caregiver assumes they know how I'm feeling without asking me directly or taking time to talk with me.	✓	O
		My primary caregiver often compares me to other people, such as siblings, relatives, or peers.	✓	✓
		When we experience conflict, my primary caregiver tends to withdraw or stop communicating with me.	O	✓
		My primary caregiver supports me in solving problems I encounter.	✓	✓
4. Cultural expectations and identity expression in the family	“*I would also say that even where you are from in Latin America influences this, at least for me, because my father is Cuban. My mother's Colombian, and I feel like if he was Colombian, then machismo would be much less.” * *“…Sometimes I just want to be alone because in Latino families you're always there with each other, very close knit. But sometimes it can be too much, and you just want to go to your room, have the closed door. That's also another thing. It can be very overbearing.” * *“Sometimes parents could nag you about–sometimes it has to do with looks or the way you dress or the way you present yourself…But my parents would be like, ‘Oh, but this is how you're supposed to look, and it'd just be back and forth, constant nagging…”*	Spending time with my family doesn't feel emotionally draining or overwhelming.	X	—
		Sometimes when I spend time with my family, I feel like I have to change how I act or dress.	O	✓
		How often do you feel pressured by your family to participate in activities that you personally do not want to engage in (e.g., attending religious services, family gatherings, cultural traditions)?	O	✓
5. Parental involvement, monitoring, and shared family life	“*…Not really asking the quality of the time you spend.”*	My parents show interest in knowing who my close friends are and who I spend time with.	O	✓
	“*Yeah. Some Hispanic parents will see-like I had some friends that do outwardly–are very comfortable with their sexuality and their gender, and a lot of parents could be like, ‘Oh, don't hang around that person, I don't want you to end up like them.' And then you feel bad in yourself, and you feel like maybe I shouldn't be this or you just end up feeling guilty. And then an uncomfortable situation of, ‘Do I still want to hang out with my friend, or do I want to get in trouble with my parents?”'*	My parent sometimes asks questions about my friends' families that feel inappropriate or intrusive.	X	—
		My parent doesn't usually go out of their way to get to know my close friends' parents.	X	—
		My parent asks how I'm being treated at school and whether I'm treated the same as other students.	X	—
		I am often allowed to choose the activities I do with my primary caregiver.	X	—
	“*And then for, let's say, if your parent disapproves of something you do, it could be like, oh, they take away your phone, they punish you, they use harmful words, or they– how would they treat the situation?” * *“So, I feel like they listen more when we're talking about school and how it went or what I got on a test more so than how I might feel in terms of sexual orientation.”*	I enjoy the activities I do with my primary caregiver.	X	—
		The time I spend with my primary caregiver feels meaningful and helps strengthen our relationship.	O	✓
		Problems or arguments often occur when I do activities or go out with my primary caregiver.	✓	✓
When my primary caregiver disapproves of something I do, they respond in a way that feels harsh or emotionally hurtful (e.g., punishment, taking away privileges, using harmful words).		X	—

X, item removed; O, item modified and retained; ✓, item retained.

Items modified in Phase 1 and subsequently retained in Phase 2 reflects the updated, modified item.

#### Theme 1: Navigating communication about sexual orientation in a cultural context

Communication about sexual orientation was frequently described as constrained by Hispanic cultural norms that positioned conversations about sex, sexual orientation, and relationships as taboo within the family. Many participants noted that these topics were typically avoided, creating uncertainty about how caregivers might react and contributing to discomfort when adolescents considered raising them. The anticipated awkwardness of disagreement, particularly within the context of “Latin parents,” often led to embarrassment and hesitation, even in cases where the eventual conversation was more neutral than expected. Participants also emphasized substantial variability across families, with some describing households where these topics could be discussed openly or with humor, underscoring that cultural expectations interact with individual family climates.

#### Theme 2: Emotional safety, self-censorship, and selective disclosure

Participants described communication with caregivers as shaped by a need to carefully manage what they share, reflecting concerns about negative reactions and respect within the family. Participants distinguished between topics they felt comfortable discussing and those they avoided, noting that even when a topic could be introduced, the specific language used required caution. This led to a pattern of self-monitoring, where adolescents were “careful” about what they said and what they chose to withhold. Many described turning to peers for support, perceiving friends as more understanding and less reactive than parents, particularly for sensitive issues.

#### Theme 3: Caregiver understanding, validation, and misattunement

A recurring concern among participants was the gap between being heard and being meaningfully understood by caregivers. Although caregivers were often perceived as attentive, adolescents noted that attentiveness did not consistently translate into accurate understanding of their emotional experiences. Responses that questioned the legitimacy of adolescents' feelings, minimized their concerns, or included unintentionally hurtful remarks contributed to a sense of being dismissed or invalidated. Over time, these patterns shaped adolescents' perceptions of caregiver responsiveness, even in relationships otherwise characterized by care.

#### Theme 4: Family reactions to identity and expression

Family dynamics were described as deeply influenced by culturally grounded expectations regarding behavior, appearance, and family closeness. Participants noted that the close-knit nature of Hispanic families could feel both supportive and overwhelming, particularly when constant proximity limited opportunities for privacy or autonomy. Cultural expectations also extended to self-presentation, with adolescents describing pressure to conform to specific norms about how they were “supposed to look” or behave. These expectations often led to tension, particularly when they conflicted with adolescents' sense of identity, resulting in ongoing negotiation between individual expression and family norms.

#### Theme 5: Parental involvement, social regulation, and connection within strain

Adolescents described family involvement as encompassing both monitoring and shared daily interactions, which were experienced as simultaneously supportive and restrictive within the context of their identity. Caregivers' efforts to stay informed about adolescents' social lives, particularly friendships, were often interpreted as protective, yet could also become a source of tension when peers were perceived as influencing sexual orientation. Participants noted that this monitoring sometimes extended into judgments about who they should associate with, leading to feelings of guilt and internal conflict. At the same time, everyday family interactions, such as discussing school or spending time together, reflected an emphasis on involvement that did not always extend to adolescents' identity-related experiences. Disapproval of behaviors was often expressed through harsh or punitive responses, further shaping how adolescents navigated their relationships with caregivers.

### Phase 2 modified Delphi panel

A table summarizing items retained, modified, or dropped throughout modified Delphi process can be found in Table 3 and a figure providing an overview of the process can be found in Figure 1.

**Figure 1:**
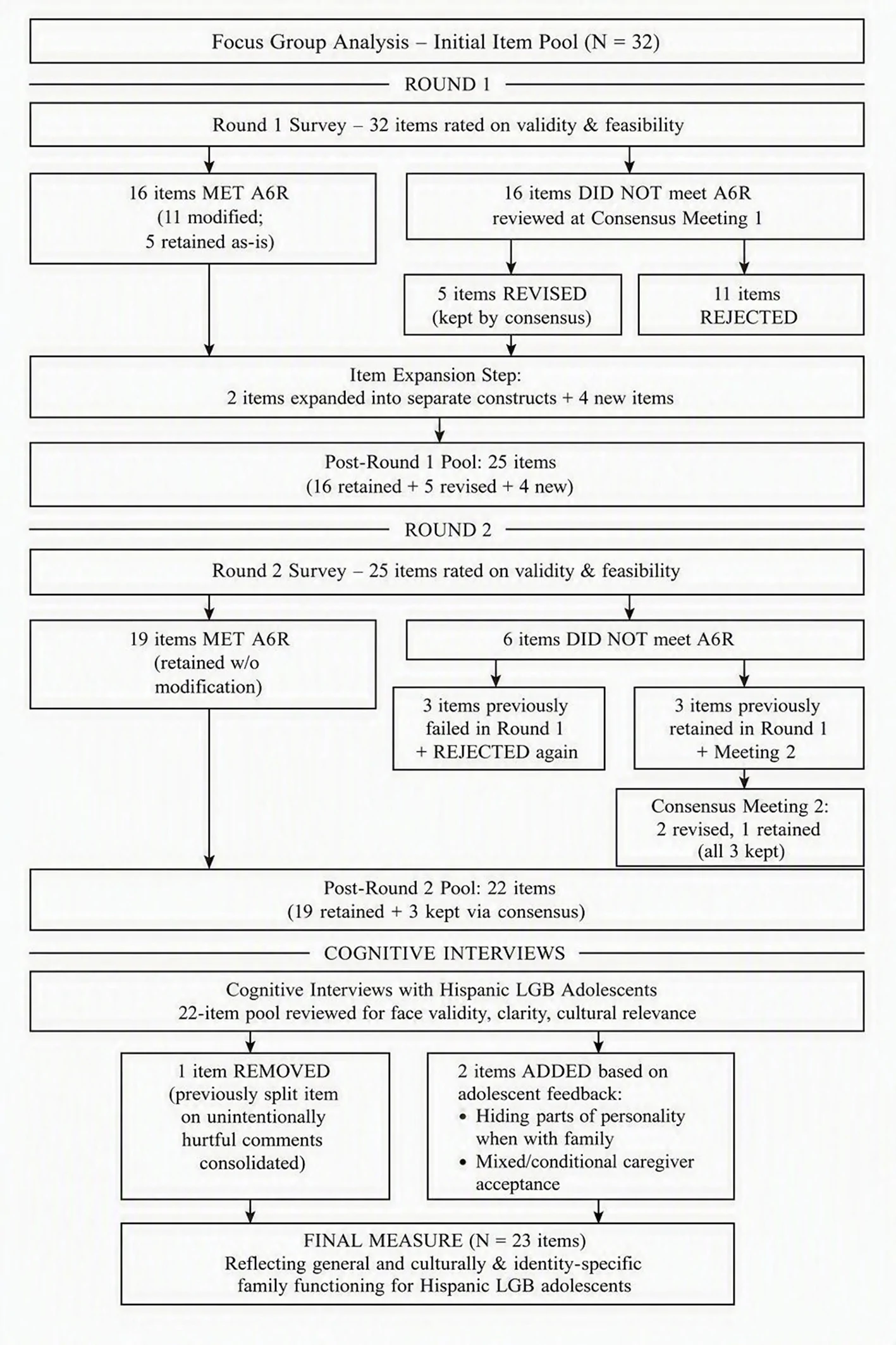
Diagram of modified Delphi process.

#### First survey and consensus meeting 1

Following analysis of focus group data, an initial pool of 32 candidate items was generated. Expert panelists rated each item on validity and feasibility, with median validity and feasibility ratings of 7.00 (ranges: 5.50–8.00 for both). General panel feedback included: using one subject and one verb per item, avoiding reverse or inconsistently worded items, and ensuring that each item reflected a single underlying construct. Of the 32 items, 16 met the A6R criterion (i.e., ratings within ±3 of the median) and were retained, however 11 of the 16 were modified.

The 16 items that did not meet the A6R criterion were reviewed during the panel meeting; of these, five were revised based on group consensus and 11 were rejected with agreement.

Additionally, two items were expanded into separate constructs, resulting in four new items. The final item pool following the first survey and consensus meeting consisted of 25 items.

#### Second survey and consensus meeting 2

Following the first consensus meeting, 25 items were advanced to Round 2 for further evaluation. Expert panelists again rated each item on validity and feasibility, with median ratings of 7.00 for both (ranges: 6.50–8.00 for validity and 6.00–8.00 for feasibility). Of the 25 items, 19 met the A6R criterion and were retained, while six were excluded. Of the six items, three items that had been excluded in the first round were excluded again, and three items retained in Round 1 were subsequently excluded in Round 2. The second meeting focused on the three items retained in Round 1 that were excluded in Round 2; two were further revised and one was retained, resulting in a final pool of 22 items following the second consensus meeting.

### Phase 3 cognitive interviews

The interview participants were, on average, 16.7 years old (*SD* = *1.5*). Most participants were assigned female at birth (76.9%). In terms of gender identity, nine participants identified as female (69.2%), two as male (15.4%), and two as transgender (15.4%). The majority of the interview participants were born in the United States (76.9%), while the remaining participants were born in Latin American countries (e.g., Nicaragua and the Dominican Republic). Reported sexual orientation was diverse: two participants described themselves as gay (15.4%), six as lesbian (46.2%), and four as bisexual (30.8%). One participant reported they had not figured out or were in the process of figuring out their sexuality (7.7%). Full participant demographics can be found in Table 1.

Two themes emerged from the cognitive interviews. First, participants consistently described the refined family functioning items as clear, understandable, and highly relevant to their lived experiences, indicating strong evidence of face validity. Second, participants offered suggestions that pointed to ways the measure could better capture identity expression and conditional acceptance within the family context. Illustrative quotes and corresponding item modifications and additions can be found in Table 4.

**Table 4:** Cognitive interview themes, illustrative quotes, and corresponding item edits.

Theme	Illustrative quotes	Corresponding item edits
1. Strong face validity and relevance of items	“*Again, these are pretty easy to understand, and I think they're, I think they're good. You know? I feel like, – yeah, I feel like I understand them pretty easily, and they're, they're worded right, if that makes sense.” * *“They're definitely, um, centered around, like, um, how the – how the primary caregiver, um, reacts to situations regarding to, um, like, their own priorities and during conflicts, especially if they tend to bring up certain, like, issues. Because I know some parents or caregivers tend to do that, especially if they tend to bring up issues regarding the – our sexual orientation.” * *“…I think these questions definitely, like, nail the, the exact feelings that we all have.” * *“…that's very, like, hands-on. The question is true. Like, I feel like a lot of people can relate to that one.”*	—
2. Refining the measurement of identity expression and conditional acceptance	“*Number 18, when it says, when I spend time with my family, I feel like, I have to change my appearance. It could also – there could also be a question about, when I had to – I feel like I have to change my personality.”*	*My primary caregiver makes comments toward me that they may not intend to be hurtful, but make me feel misunderstood*.
		^*^I feel like I have to hide parts of my personality when I am with my family.
	“*This one relates a bit more to the LGBT topic, but it still feels a little off-topic. That's just my opinion. Maybe they could specify that the conflict is about my sexual orientation, like: ‘When we have a conflict about my orientation, my primary caregiver tends to distance themselves.' Something like that.” * *“…they could make it clearer by adding something like ‘about my orientation,' so it becomes more specific.” * *“But, like, do you feel that your parent has some kind – even when they tell you that they are accepting, do you feel that they have some kind of prejudice against you, uh, whether, whether or not they even know it themselves? Um, I don't know how you would word that, but something like that.”*	^*^My primary caregiver supports me in some ways but not in others when it comes to who I am.
	I feel afraid to ask my primary caregiver for what I need **regarding my sexual orientation**.
	When we experience conflict **about my sexual orientation**, my primary caregiver tends to withdraw.

Item edits are bolded. Removed items are noted in italics. New items are noted with an asterisk.

#### Theme 1: Strong face validity and relevance of items

Overall, participants responded positively to the refined family functioning items. Across interviews, they emphasized that the items were clear, easy to understand, and relevant to their experiences as Hispanic LGB adolescents. Many participants also offered concrete personal examples that illustrated how the items reflected real family dynamics, suggesting strong face validity and perceived relevance.

#### Theme 2: Refining the measurement of identity expression and conditional acceptance

Although most participants affirmed the wording and content of the items, they also provided several useful suggestions for refinement. One recurring recommendation was to broaden the item “When I spend time with my family, I feel like I have to change my appearance” to better capture internal and behavioral self-presentation, not just visual appearance. Participants noted that family interactions may require changes in how one acts or carries oneself, and one participant explained that some adolescents may feel pressure to appear “more masculine” when around family, even if that change is not visible in appearance alone. In response to this feedback, participants suggested adding “personality” to the item or adding a new item to reflect both outward appearance and internal or behavioral changes.

Participants also identified a need for items assessing a disconnect between stated parental acceptance and enacted behavior. Several noted that a parent may claim to be accepting while still making homophobic comments, failing to defend the adolescent in front of other family members, or otherwise behaving in ways that feel inconsistent with acceptance.

Relatedly, participants suggested that the measure should capture the adolescent's own sense that a parent is not genuinely accepting, even when acceptance is verbally expressed. Participants additionally proposed that some items could benefit from additional specificity related to sexual orientation. For example, they recommended assessing whether conflict experienced with caregivers was conflict related to sexual orientation.

### Final item refinements

Following analysis of cognitive interview data, which presented the 22 items agreed upon in the second Delphi consensus round, we removed one item that had been separated into two separate items (“*My primary caregiver makes comments towards me that they may not intend to be hurtful, but make me feel misunderstood*.”), resulting in 21 items. Based on feedback for the item: “*When I spend time with my family, I feel like I have to change my appearance*” we added an additional item (item 22) “*I feel like I have to hide parts of my personality when I am with my family*.” We also added an additional item (item 23) “*My primary caregiver supports me in some ways but not in others when it comes to who I am*” based on adolescent feedback regarding varying levels of acceptance among parents. To further refine existing items, we modified the item “*I feel afraid to ask my primary caregiver for what I need”* to “*I feel afraid to ask my primary caregiver for what I need regarding my sexual orientation*.” Similarly, the item “*When we experience conflict, my primary caregiver tends to withdraw*” was modified to “*When we experience conflict about my sexual orientation, my primary caregiver tends to withdraw*.” These changes were reviewed and approved by the expert panel. The final item pool was translated into Spanish and back-translated into English following procedures outlined by Kurtines and Szapocznik (47) and can be found in Table 5.

**Table 5:** Final items in English and Spanish.

English	Spanish
I feel comfortable talking to my primary caregiver about my sexual orientation.	Me siento cómodo/a hablando con mi cuidador/a principal sobre mi orientación sexual.
My primary caregiver always believes what I am saying regarding my sexual orientation.	Mi cuidador/a principal siempre me cree cuando hablo sobre mi orientación sexual.
I feel afraid to ask my primary caregiver for what I need regarding my sexual orientation.	Siento miedo de pedirle a mi cuidador/a principal lo que necesito en relación con mi orientación sexual.
My primary caregiver tends to focus more on their own priorities (e.g., what/who they want me to be, or become) than on what matters to me.	Mi cuidador/a principal tiende a enfocarse más en sus propias prioridades (por ejemplo, lo que quiere que yo sea o en quien quiere que me convierta) que en lo que es importante para mí.
When we experience conflict about my sexual orientation, my primary caregiver tends to withdraw.	Cuando tenemos conflictos sobre mi orientación sexual, mi cuidador/a principal tiende a alejarse.
When we experience conflict, my primary caregiver tends to stop communicating with me.	Cuando tenemos conflictos, mi cuidador/a principal tiende a dejar de comunicarse conmigo.
I'm careful about what I say to my primary caregiver so I don't upset them.	Tengo cuidado con lo que le digo a mi cuidador/a principal para no molestarle/la.
There are certain things I don't tell my primary caregiver because I don't want them to find out.	Hay cosas que no le cuento a mi cuidador/a principal porque no quiero que se entere.
When I express my feelings, my primary caregiver makes me feel uncomfortable.	Cuando expreso mis sentimientos, mi cuidador/a principal me hace sentir incómodo/a.
I avoid discussing certain topics, such as my sexual orientation, with my primary caregiver.	Evito hablar de ciertos temas, como mi orientación sexual, con mi cuidador/a principal.
My primary caregiver supports me in solving problems I encounter.	Mi cuidador/a principal me apoya para resolver los problemas que enfrento.
My primary caregiver makes comments about LGB people that upset me.	Mi cuidador/a principal hace comentarios sobre personas LGB que me molestan.
My primary caregiver makes comments toward me that they may not intend to be hurtful, but that make me feel misunderstood.	Mi cuidador/a principal hace comentarios hacia mí que quizás no pretende que sean hirientes, pero que me hacen sentir incomprendido/a.
My primary caregiver assumes they know how I'm feeling without asking me directly	Mi cuidador/a principal asume que sabe cómo me siento sin preguntarme directamente.
My primary caregiver shows interest in knowing who my close friends are.	Mi cuidador/a principal muestra interés en conocer quiénes son mis amigos/as cercanos/as.
My primary caregiver shows interest in knowing who I spend time with.	Mi cuidador/a principal muestra interés en saber con quién paso el tiempo.
When I spend time with my family, I feel like I have to change my appearance.	Cuando paso tiempo con mi familia, siento que tengo que cambiar mi apariencia.
I feel pressured by my family to participate in activities that I do not want to engage in (e.g., attending religious services, family gatherings, cultural traditions)?	¿Siento presión por parte de mi familia para participar en actividades que no quiero realizar (por ejemplo, asistir a servicios religiosos, reuniones familiares o tradiciones culturales)?
My primary caregiver compares me to other people, such as siblings, relatives, or peers.	Mi cuidador/a principal me compara con otras personas, como hermanos/as, familiares o compañeros/as.
The time I spend with my primary caregiver feels meaningful.	El tiempo que paso con mi cuidador/a principal se siente significativo.
Problems or arguments occur during activities with my primary caregiver.	Surgen problemas o discusiones durante las actividades con mi cuidador/a principal.
My primary caregiver supports me in some ways but not in others when it comes to who I am.	Mi cuidador/a principal me apoya en algunos aspectos pero no en otros cuando se trata de quién soy.
I feel like I have to hide parts of my personality when I am with my family.	Siento que tengo que ocultar partes de mi personalidad cuando estoy con mi familia.

Response options range from 1 = strongly disagree to 5 = strongly agree. All options will include a “not applicable” response for items contingent on experiences that may not have occurred.

## Discussion

The purpose of this study was to iteratively develop a user-informed, culturally responsive measure of family functioning for Hispanic LGB adolescents. The measure was developed following a multi-phase design: (a) focus groups with Hispanic LGB adolescents to identify salient family functioning processes; (b) a multi-round modified Delphi panel with community partners and experts in measure development for Hispanic and LGB populations to refine items, establish consensus on relevance and clarity, and ensure alignment with theoretical constructs; and (c) cognitive interviews to assess the face validity of the developed measure with Hispanic LGB adolescents. The resulting 23-item pool reflects both general family functioning processes impacted by disclosure of sexual orientation and LGB-specific culturally shaped experiences.

Focus group findings indicate that family functioning for Hispanic LGB adolescents is context-dependent and shaped by culturally influenced processes related to sexual orientation. Communication emerged as the primary mechanism through which adolescents navigated disclosure, with decisions to share or withhold guided by anticipated parental reactions, consistent with prior work (22). These findings also align with the Disclosure Process Model (48) which conceptualizes disclosure as shaped by approach goals, such as seeking authenticity, connection, or support, and avoidance goals, such as preventing rejection, conflict, or other negative reactions. Consistent with broader disclosure literature, adolescents' selective disclosure and careful management of what they shared with caregivers appeared particularly responsive to anticipated reactions, while peers sometimes provided alternative sources of connection and support (49). These processes were closely tied to adolescents' perception of their parents' comfort and responsiveness, as well as to whether parents moved beyond *listening* toward meaningful understanding of adolescents' experiences. Cultural values further shaped how adolescents navigated identity expression and family roles, contributing to tension between individual identity and expectations for family harmony (4, 22). Adolescents also described parent involvement as encompassing support, monitoring, and regulation, often experienced simultaneously.

With this information, our team developed an initial pool of 32 candidate items for the first consensus meeting, which were evaluated by a panel of community partners and measurement experts to refine items for clarity, feasibility, and cultural congruence while preserving theoretically important content identified by Hispanic LGB adolescents. Revisions emphasized clarity and face validity by simplifying wording and ensuring each item reflected a single, distinct concept. Items assessing more than one content area in a single item (e.g., double-barreled items) were refined or separated to improve interpretability and wording used one subject and one verb per item. Additional modifications increased specificity (e.g., limiting comparisons to siblings) and removed redundant or additive phrasing (e.g., “helps strengthen our relationship”) to reduce ambiguity. In the second consensus meeting, two items were further refined to improve clarity. Consensus across academic experts and community partners incorporated diverse perspectives, enhancing the cultural and developmental appropriateness of the final item set.

Cognitive interview findings provided important additional support for the measure's face validity while offering targeted refinements to strengthen item precision. Participants consistently viewed the items as clear and reflective of their experiences, reinforcing that the measure captured meaningful aspects of family functioning. At the same time, their feedback highlighted areas where greater nuance was needed, particularly in capturing identity expression beyond appearance and distinguishing between verbal and enacted acceptance. Revisions addressed these gaps by incorporating items that reflect behavioral and internal self-presentation, as well as discrepancies between stated support and lived experiences. Participants also suggested making certain items more explicitly tied to sexual orientation, improving alignment with the construct of interest. Taken together, these suggestions indicated that while the items were generally viewed as understandable and culturally appropriate, participants identified several ways to improve their sensitivity to the complexity of family acceptance and sexual orientation among Hispanic LGB adolescents. It is important to note that 9 of the 13 adolescents who participated in the Phase 3 cognitive interviews had previously participated in Phase 1. Prior familiarity with the study and its focus on family functioning may have influenced participants' evaluations of the items and contributed to the strong face-validity findings; therefore, replication with an independent sample is needed to confirm relevance and comprehensibility.

### Implications

The development of this measure has several implications for public health research, practice, and future measurement work with Hispanic LGB adolescents. Most broadly, the findings reinforce that family functioning is not a generic construct that can be assumed to operate the same way across populations, especially when sexual orientation, cultural expectations, and adolescent development intersect. The measure also responds to recent evidence that existing family-functioning measures may not function equivalently across Hispanic LGB and heterosexual adolescents and may not always appear face valid to Hispanic LGB adolescents (25, 26). Accordingly, the present work contributes to a more precise understanding of how family functioning should be conceptualized and assessed in studies of Hispanic LGB adolescents.

#### Public health research and practice

For research, this measure offers a more tailored tool for examining family functioning as a mechanism of risk and resilience among Hispanic LGB adolescents. Prior work has shown that family acceptance, rejection, communication, and involvement are linked to adolescent HIV- related outcomes, drug use, and mental-health problems (16, 18, 19). However, these links are likely to operate differently when family dynamics are shaped by both sexual orientation and culturally specific expectations about family roles, loyalty, and communication (4, 22). This measure allows researchers to test whether family functioning predicts mental health outcomes over and above more general parenting constructs and whether it mediates or moderates associations between disclosure, minority stress, and health outcomes.

This measure may also support intervention research by offering an outcome or process measure closely aligned with the family experiences of Hispanic LGB adolescents. This is particularly important given that family-based interventions for this population increasingly target family communication, support, and involvement, yet many studies continue to rely on generic family measures that may not fully capture intervention-relevant change (50–52). This measure can therefore elucidate how family functioning shapes health outcomes and to identify intervention targets and mechanisms addressing co-occurring HIV-related outcomes, drug use, and mental-health problems among Hispanic LGB adolescents.

Finally, the measure may help clinicians, school-based providers, and family interventionists identify the areas of family functioning most salient for Hispanic LGB adolescents. A culturally responsive measure can help providers move beyond broad, binary impressions of “good” or “bad” parenting and instead assess more specific domains such as communication about sexual orientation, caregiver involvement, and reactions to disclosure. This can support more targeted conversations with families and may help providers recognize when verbal acceptance is not matched by behavior.

#### Measurement

The developed measure addresses a major gap in existing family functioning tools, which often omit disclosure-related, identity-specific, and culturally specific family processes relevant to Hispanic LGB adolescents. The iterative design process increases the likelihood that the final measure will be understandable, acceptable, and conceptually relevant to the target population given that content validity has been established among Hispanic LGB adolescents themselves and experts (i.e., researchers and community partners). Future directions include psychometric evaluation in a larger sample to empirically determine the measure's dimensionality and whether the items support one or more subscales and/or an overall family functioning score. Additionally, future work will evaluate the reliability of the measure in addition to examining the concurrent and predictive validity of the measure in relation to HIV-related outcomes, drug use, and mental-health problems. Moreover, to promote equitable measurement and minimize bias, advanced methods such as moderated nonlinear factor analysis can be used to assess differential item functioning across subgroups, allowing for the identification and adjustment of items that function differently across populations and ensuring that the measure more accurately reflects diverse experiences (53, 54).

### Limitations and strengths

The study findings should be interpreted while considering the following limitations.

First, the sample was drawn from Hispanic LGB adolescents who were willing to participate in a research study and discuss family experiences, and minors were required to have disclosed their sexual orientation to one parent as parental consent for participation is required in the state of Florida. Although participants described a wide range of family dynamics and levels of acceptance, this requirement excluded adolescents who had not disclosed their sexual orientation, including those who may have anticipated rejection, conflict, or other negative family reactions. Consequently, adolescents navigating nondisclosure or particularly unsupportive family environments may be underrepresented in the resulting item pool. Future research should evaluate the measure among Hispanic LGB adolescents who have not disclosed their sexual orientation and determine whether additional family functioning experiences or items emerge. Relatedly, although some participants identified as transgender or gender-fluid, eligibility was based on sexual orientation, and the measure was developed to assess family functioning in relation to sexual orientation rather than gender identity. Thus, the items may not fully capture family processes specific to gender identity or expression, and future research should evaluate and adapt the measure for transgender and gender-diverse adolescents.

Second, the relatively small sample was recruited exclusively from Miami-Dade County, limiting generalizability to Hispanic LGB adolescents in other geographical and cultural contexts. The Hispanic population is heterogeneous, and our sample may not adequately represent variation by national origin, immigration history, or acculturation. Indeed, participants themselves noted that family dynamics may vary by national origin (see Table 2, Theme 4), underscoring the importance of examining within-group differences in future research. Third, the modified Delphi panel was relatively small (*N* = 6), although this panel size is consistent with prior measure-development studies in this area (36, 37). Finally, this study focused solely on adolescents' perspectives of family functioning and did not include caregiver reports, which may differ and have been shown to overestimate family functioning relative to adolescents reports (55).

Despite these limitations, this study has several notable strengths. First, it employed a user-centered, multi-phase development process that integrated the perspectives of Hispanic LGB adolescents, community partners, and measurement experts. Second, the study intentionally focused on a population for whom existing family functioning measures may be insufficient, increasing the cultural and clinical relevance of the resulting instrument. Third, the inclusion of cognitive interviews enabled iterative refinement of item wording based on adolescents' lived experiences, enhancing both the clarity and contextual relevance of the final measure.

## Conclusion

In conclusion, this study advances the measurement of family functioning for Hispanic LGB adolescents by developing a measure that is grounded in adolescents' lived experiences and refined through iterative feedback from adolescents, community partners, and measurement experts. The resulting item set captures family processes that are especially relevant to this population, including communication about sexual orientation, monitoring, involvement, and the complexity of acceptance and support within Hispanic families.

More broadly, this measure addresses a critical gap in the assessment literature by moving beyond generic family functioning tools that may not adequately reflect the cultural and developmental realities of Hispanic LGB adolescents. By offering a more contextually responsive instrument, this work lays the foundation for future research on risk and resilience, for stronger evaluations of family-based interventions, and for more precise clinical and community assessment. The next phase of this work will further evaluate the measure's psychometric properties in a larger sample, strengthening its potential utility for researchers and practitioners seeking to understand and support the health and wellbeing of Hispanic LGB adolescents.

## Data Availability

The raw data supporting the conclusions of this article will be made available by the authors, without undue reservation.
