# Supplementary material for: Development of a family functioning measure for Hispanic lesbian, gay, and bisexual adolescents
Source: Front Public Health. 2026 Oct 7;14:1970713. doi: 10.3389/fpubh.2026.1970713 (PMC13683103; doi:10.3389/fpubh.2026.1970713)
Supplement: Supplementary file 1 [file Data_Sheet_1.pdf]

Supplemental Tables

Supplemental Table 1

Focus Groups Question Guide

Question
1. How does your parents' Hispanic culture, including attitudes towards sexual orientation, impact your relationship with them?
2. Are there certain topics, including those related to sexual orientation, that you avoid talking to your parent about because of their cultural values?
3. What role does family, including acceptance of diverse sexual orientations, play in Hispanic culture, and how does this affect the relationship between you and your parent?
4. How do expectations around gender roles, as well as expectations regarding sexual orientation, affect the relationship between you and your parent?
5. What else is important to capture about HSMA's relationships with their parent(s), particularly regarding acceptance and understanding of sexual orientation within the Hispanic cultural context?

Supplemental Table 2

Individual Interview Question Guide

Question
1. What did that question mean to you?
2. Was it easy to understand, or did you think it might have meant something else?
3. Was it easy to decide which response to choose?
4. What is missing from these questions?
5. Based on your experience, are any of these domains not relevant for you?
6. What kind of questions would be more relevant for youth from your culture?
7. What else about family is important to capture in these questions?

Supplemental Table 3

Delphi Panel Question Guide

Question
1. How well do you think the language in these items reflects the cultural nuances and family dynamics experienced by Hispanic sexual minority adolescents?
2. Can you describe any aspects of family functioning unique to HSMA that you feel might be missing from these measures?
3. In what ways could the wording of these items be adjusted to be more inclusive and clear for adolescents?
4. How do you see these items capturing the impact of a youth's sexual orientation disclosure on overall family functioning?
5. What suggestions do you have for integrating cultural context and family history more effectively into these measures?
6. How might these items be refined to better identify specific areas where intervention or support is needed for HSMA?
